# New Insights on the Vibrational Dynamics of 2-Methoxy-, 4-Methoxy- and 4-Ethoxy-Benzaldehyde from INS Spectra and Periodic DFT Calculations

**DOI:** 10.3390/ma14164561

**Published:** 2021-08-13

**Authors:** Paulo J. A. Ribeiro-Claro, Pedro D. Vaz, Mariela M. Nolasco, Francisco P. S. C. Gil, Luís A. E. Batista de Carvalho, Maria Paula M. Marques, Ana M. Amado

**Affiliations:** 1 CICECO, Departamento de Química, Universidade de Aveiro, 3810-193 Aveiro, Portugal; prc@ua.pt; 2 Champalimaud Centre for the Unknown, Champalimaud Foundation, 1400-038 Lisboa, Portugal; pedro.vaz@fundacaochampalimaud.pt; 3 Departamento de Física, Universidade de Coimbra, 3004-516 Coimbra, Portugal; fgil@uc.pt; 4 Química-Física Molecular, Departamento de Química, Universidade de Coimbra, 3004-535 Coimbra, Portugal; labc@ci.uc.pt (L.A.E.B.d.C.); pmc@ci.uc.pt (M.P.M.M.); amadoam@gmail.com (A.M.A.); 5 Departamento de Ciências da Vida, Universidade de Coimbra, 3000-456 Coimbra, Portugal

**Keywords:** density functional theory, inelastic neutron scattering, vibrational assignment, molecular crystal, torsional potential, C–H…O hydrogen bonds

## Abstract

The dynamics of 2-methoxybenzaldehyde, 4-methoxybenzaldehyde, and 4-ethoxybenzaldehyde in the solid state are assessed through INS spectroscopy combined with periodic DFT calculations. In the absence of experimental data for 4-ethoxybenzaldehyde, a tentative crystal structure, based on its similarity with 4-methoxybenzaldehyde, is considered and evaluated. The excellent agreement between calculated and experimental spectra allows a confident assignment of the vibrational modes. Several spectral features in the INS spectra are unambiguously assigned and torsional potential barriers for the methyl groups are derived from experimental frequencies. The intramolecular nature of the potential energy barrier for methyl rotation about O–CH_3_ bonds compares with the one reported for torsion about saturated C–CH_3_ bonds. On the other hand, the intermolecular contribution to the potential energy barrier may represent 1/3 of the barrier height in these systems.

## 1. Introduction

In the last years, there has been an increasing number of reports assessing the structure and dynamics of molecular crystals based on the synergistic combination of inelastic neutron scattering (INS) spectra with periodic density functional (DFT) calculations (e.g., [1,2,3,4,5,6,7,8,9]). In INS, there are no selection rules and band intensities are proportional to the nuclei scattering cross-section and atomic displacements in the vibrational mode, both of which are large for hydrogen atoms. In this way, INS spectroscopy provides information not accessible using optical vibrational techniques (IR and Raman) and presents high sensitivity for low wavenumber/large amplitude vibrations, such as torsional vibrations and molecular librational and translational modes. On the other hand, DFT calculations—either periodic or discrete—are highly efficient in predicting the eigenvectors (atomic displacements) for the vibrational normal modes, all that is required to simulate the corresponding INS spectrum. The combined use of INS spectroscopy and periodic DFT calculations has been reported by us for several systems, including vegetable and bacterial cellulose [1], furandicarboxylate-based polymer [2], and benzaldehyde derivatives [4,9].

An additional advantage of this combined approach is that it can be used to validate proposed structures, when the experimental structural data is missing. This is particularly valuable for amorphous systems, for which the experimental INS spectrum can be matched to a combination of proposed model structures whose spectral contributions are determined from discrete DFT. This methodology has been comprehensively described in the influential work on doped poly(hexylthiophene) [10] and it has been used by some of us to experimentally assess the random nature of 2,4-poly(ethylene furanoate) [11] and to interpret the structure of bacterial cellulose composites [12]. It can also be advantageous to validate proposed crystal structures, namely those derived from periodic DFT calculations. In the absence of the experimental crystal structure (e.g., X-ray or neutron diffraction), an optimized structure can be used to generate frequencies and intensities, and from that the corresponding INS spectrum. If the simulated INS spectrum obtained from the optimized “crystal structure” fails to adequately represent the experimental INS spectra, the structure should be rejected.

**Figure 1:**
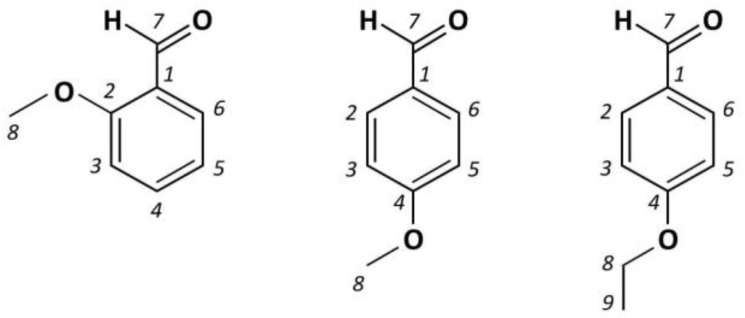
Schematic representation of the 2-methoxy, 4-methoxy, and 4-ethoxybenzaldehyde molecules. The molecules display a planar heavy-atom skeleton and adopt the anti-conformation (C=O and O–C(8) bonds pointing to opposite sides of the ring).

In this work, the periodic DFT approach is applied to get new insights on the vibrational dynamics of three benzaldehyde derivatives in the crystalline form whose INS spectra have been recorded in the absence of an experimental crystal structure: 2-methoxybenzaldehyde, 4-methoxybenzaldehyde, and 4-ethoxybenzaldehyde (Figure 1). The crystal structure of 2-methoxybenzaldehyde had been obtained earlier by some of us [13]. More recently, the crystal structure of 4-methoxybenzaldehyde has been reported from X-ray diffraction [14], thus providing the information required for a periodic DFT simulation of INS spectrum. For 4-ethoxybenzaldehyde, in the absence of experimental data, a proposed crystal structure, optimized by periodic DFT based on the similarity with 4-methoxybenzaldehyde, is considered and evaluated. In all cases, the low wavenumber region of the spectra is given particular attention. The presence of band splitting and the dynamics of methyl torsion in the crystal are evaluated and discussed.

## 2. Materials and Methods

Compounds: 2-methoxybenzaldehyde (2MeOB), 4-methoxybenzaldehyde (4MeOB), and 4-ethoxybenzaldehyde (4EtOB) were obtained commercially and used as supplied.

INS spectroscopy: Inelastic neutron scattering (INS) experiments were performed with the TOSCA spectrometer, an indirect geometry time-of-flight spectrometer at the ISIS Neutron and Muon Source at the Rutherford Appleton Laboratory (Chilton, UK) [15,16]. The samples, with a total amount of ca. 2 g, were packed inside flat thin-walled aluminum cans of 5 cm height and 4 cm width, with a path length of 2 mm, which were mounted perpendicular to the beam using a regular TOSCA centre-stick. Spectra were collected below 15 K, measured for the 16 to 8000 cm^−1^ energy-transfer range, and the resolution was ΔE/E ≈ 1.5%. Data was converted to the conventional scattering law, S(Q,ν) vs. energy transfer (in cm^−1^) using the MANTID program (version 4.0.0) [17].

Far-IR spectroscopy: The ATR/Far-IR spectrum (50–600 cm^−1^) of 4-ethoxybenzaldehyde was measured on a Bruker Optics Vertex 70 FTIR spectrometer purged by CO2-free dry air, equipped with a Bruker Platinum ATR single reflection diamond accessory, using a silicon solid-state beam-splitter and a Deuterated L-alaninedoped TriGlycine Sulphate (DLaTGS) detector with a polyethylene window. The spectrum was the sum of 128 scans, at 2 cm^−1^ resolution, and the 3-term Blackman–Harris apodization function was applied. Under these conditions, the wavenumber accuracy was better than 1 cm^−1^. The spectrum was corrected for the frequency dependence of the penetration depth of the electric field in ATR (Opus 7.5 spectroscopy software).

Quantum Chemistry calculations: Single molecule (discrete) calculations were performed with Gaussian 09 program (G09), version A.02 [18] using the PBE pure density functional combined with the 6–311G(d,p) basis set, as implemented in G09. Frequency calculations, subsequent to full geometry optimization, provide the infrared and Raman intensities and confirm the convergence to a true minimum (no imaginary frequencies). Potential energy functions for internal rotations have been obtained through the “scan” option of G09, using the step size of 10°.

Periodic density functional theory (periodic DFT) calculations were carried out using the plane-wave/pseudopotential method as implemented in the CASTEP code [19,20]. Exchange and correlation were approximated using the PBE functional [21]. The plane-wave cut-off energy was 830 eV. Brillouin zone sampling of electronic states was performed on 8 × 4 × 4 Monkhorst–Pack grid. The equilibrium structure, an essential prerequisite for lattice dynamics calculations, was obtained by LBFGS geometry optimization after which the residual forces were converged to zero within 0.005 eV·A^−1^. For 2-methoxybenzaldehyde, the initial structure was taken from the reported crystal structure [13]. For 4-methoxybenzaldehyde, the initial structure was taken from the deposited crystal structure (CSD entry: YADJIY [14]). In both cases, the cell parameters were kept constant during geometry optimization. This is important when using standard GGA functions, as their description of dispersion/van der Waals interactions is defective, leading to unrealistic cell dimensions, as discussed elsewhere [22]. For 4-ethoxybenzaldehyde, a proposed structure was built from that of 4-methoxy derivative, with the addition of one methyl group. The unit cell dimensions were increased by 63 pm and 196 pm in the directions a and c, respectively (ca. 12.6%), considering the dimension of the new methyl group and its orientation anti relative to the C–O bond (CPhO–CCMe dihedral angle set to 180°). This structure was optimized (i) with fixed cell parameters and (ii) with relaxed cell parameters, using the semi-empirical dispersion correction of Tkatchenko and Scheffler [23] to properly account for van der Waals interactions. Phonon frequencies were obtained by diagonalization of dynamical matrices computed using density-functional perturbation theory across a matrix of dimension 4 × 2 × 1 accounting for 12 q-points. In addition to the direct evaluation of frequencies and intensities at the Γ-point, the phonon dispersion was calculated along the high symmetry path throughout the Brillouin zone (BZ).

The eigenvectors (atomic displacements) for each normal mode that are part of the CASTEP and G09 output were used to visualize the modes with program Jmol [24] and to generate the INS spectrum using the program AbINS [25]. AbINS is an open-source package implemented as a plugin to the neutron data analysis software Mantid [17]. AbINS accounts for the neutron scattering cross sections, overtones and combination modes, together with instrument specific E-Q windows, to produce a calculated INS spectrum that is easily compared with experiment. It is emphasized that, for all the calculated spectra shown, the transition energies were not scaled.

The determination of the potential barrier for a single simple internal rotor from torsional transitions has been performed with the program Barrier [26].

## 3. Results

### 3.1. 2-Methoxybenzaldehyde (2MeOB)

The 2-methoxybenzaldehyde crystal structure is built up on the tetragonal space group P_43_ from an asymmetric unit composed of four independent molecules (Z = 16) [13]. The four independent crystallographic molecules are self-assembled in dimers via C–H···O hydrogen bonds between C(3)–H of one molecule and the carbonyl oxygen atom of the neighboring molecule. Table 1 compares the experimental and calculated values for a few selected parameters for the molecule labeled (A) in the reported crystal structure (see [13]). Calculated values generally agree with the experimental values and the most significant deviation is observed for the calculated C=O bond length (ca. 2 pm larger than the experimental value). This overestimation of the C=O bond length—and the concomitant underestimation of the C=O stretching frequency—is a well-known problem of the PBE functional (e.g., [6,9,22,27]), which becomes more evident in the simulation of the infrared spectrum due to the large infrared intensity of this mode.

**Figure 2:**
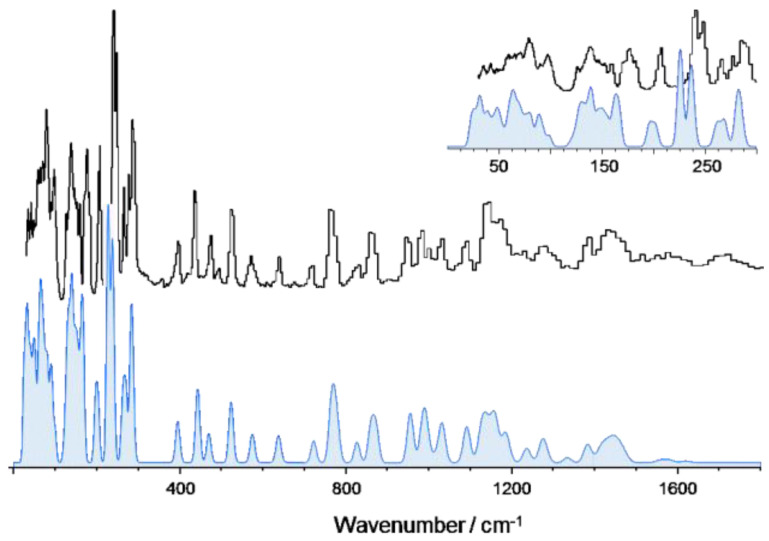
The INS spectra of 2MeOB up to 1800 cm^−1^: experimental (**top**) and simulated from periodic DFT calculations (**bottom**, CASTEP). The inset provides a detailed view of the low wavenumber region.

Figure 2, top line, presents the experimental INS spectrum of 2MeOB, in the 25–1800 cm^−1^ range. The bottom line shows the simulated INS spectrum obtained from periodic calculations (CASTEP). The corresponding full INS spectra, up to 4000 cm^−1^, and the comparison between periodic and discrete calculations at the same level are shown in Figures S1 and S2, Supplementary Material, respectively.

As it becomes obvious from Figure 2, there is a remarkable nearly one-to-one match between the calculated and observed bands. This match allows a confident assignment of the vibrational modes in the INS spectrum, which is presented in Table 2.

The infrared and Raman spectra of 2MeOB were assigned recently, with support from discrete (single molecule) DFT calculations at the B3LYP/6–311+G** level [28]. The analysis excludes the low wavenumber modes, which are more critically limited by the single molecule approach, but most of the assignments proposed seem to be consistent with those on Table 2. However, due to the use of potential energy distribution (P.E.D.) values and general descriptions of the atoms, it is not possible to fully compare the assignments.

Table 2 displays the splitting of large amplitude modes due the presence of different molecular environments in the crystal. The four independent crystallographic molecules turn out to yield two distinct vibrational transitions, related with the molecules labeled A/C and B/D [13].

### 3.2. 4-Methoxybenzaldehyde (4MeOB)

**Figure 3:**
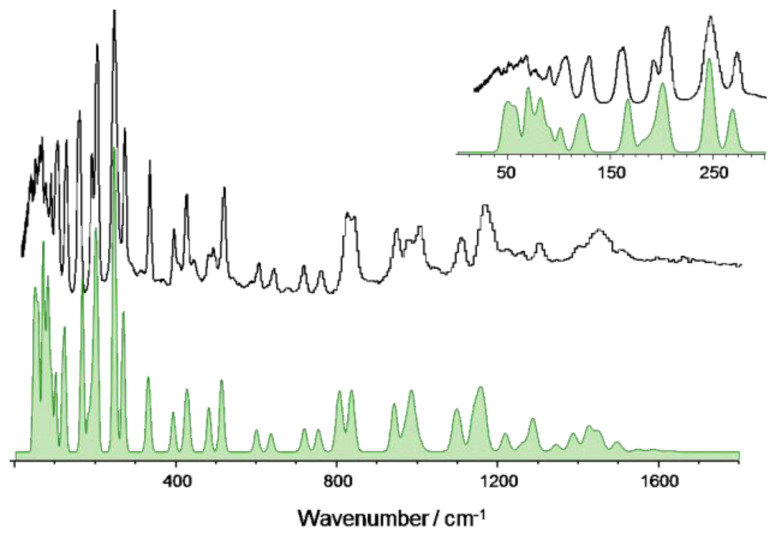
The INS spectra of 4MeOB up to 1800 cm^−1^: experimental (**top**) and simulated from periodic DFT calculations (**bottom**, CASTEP). The inset provides a detailed view of the low wavenumber region. The full INS spectra, up to 4000 cm^−1^, and the comparison between periodic and discrete calculations at the same level are shown in Figures S1 and S2, Supplementary Material, respectively.

4-methoxybenzaldehyde crystallizes in the space group P212121 with Z = 1 [14]. Molecules are arranged in zigzag chains along the c axis and are held together by weak C—H…O hydrogen bonds between the methyl group and the carbonyl group of a neighboring molecule. Periodic-DFT geometry optimization mainly preserved this structure, as shown in Table 3. As for the 2-substituted analogue, there is an excellent match between calculated and experimental INS spectra (Figure 3), supporting the assignments proposed on Table 4.

The assignment of the infrared and Raman spectra was previously reported [29,30,31]. The present results are mostly in conflict with the assignments of Gunasekaran et al. [30], except for the case of a few characteristic modes such as the C–H stretching modes. On the other hand, there is a better agreement with the assignments reported by Altun et al. for the liquid phase [31]. However, those authors did not fully discriminate the modes arising from the syn-form (C=O and O–C(8) bonds in the same side of the ring), which were observed in the liquid phase and were early identified from temperature-dependent studies [29]. In particular, the pair of bands observed at 393/371 cm^−1^, which belong to the same vibrational mode of different conformers, were assigned by Althun et al. to out-of-plane and in-plane ring deformations, respectively.

Further discrepancies between the present periodic calculations and previous discrete calculations [29,31] mostly arise for the large amplitude/low wavenumber modes. For instance, the methoxy torsional mode, τC(4)-OCH_3_, observed at 160 cm^−1^ in the INS spectrum (165 cm^−1^ in CASTEP) was previously predicted to occur around 70 cm^−1^ [29,31]. This is an example of a mode whose large amplitude motion is restricted by crystal packing and cannot be properly described by single molecule calculations. Conversely, the torsion of the more symmetrical methyl group is correctly predicted to occur near 240 cm^−1^ from both periodic and discrete calculations [29,31].

### 3.3. 4-Ethoxybenzaldehyde (4EtOB)

**Figure 4:**
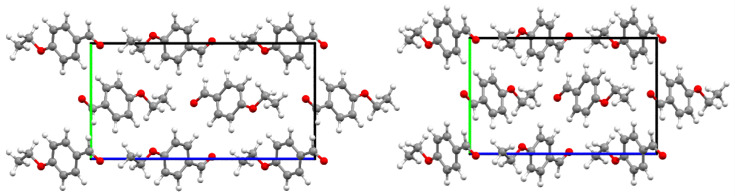
Optimized crystal structure of 4EtOB with fixed (**left**) and relaxed (**right**) cell, viewed along the “a” axis.

To the best of our knowledge, there are no reports on the crystal structure of 4-ethoxybenzaldehyde. Since the equilibrium crystal structure is a requirement to obtain the periodic DFT simulation of the INS spectrum, a tentative unit cell was built from the experimental structure of the 4-methoxybenzaldehyde analogue, and optimized with fixed and relaxed cell parameters, as described in the computational section. The optimized cell structures are shown in Figure 4, and the full data is presented in the Supplementary Material (CIF files).

Table 5 compares some selected geometrical parameters for fixed and relaxed optimized geometries. As it can be seen on Table 5, optimization of the cell parameters with DFT + Dispersion yields an expansion along the *a* axis and contraction along both *b* and *c* axes, with some relevant changes in the intermolecular distances.

**Figure 5:**
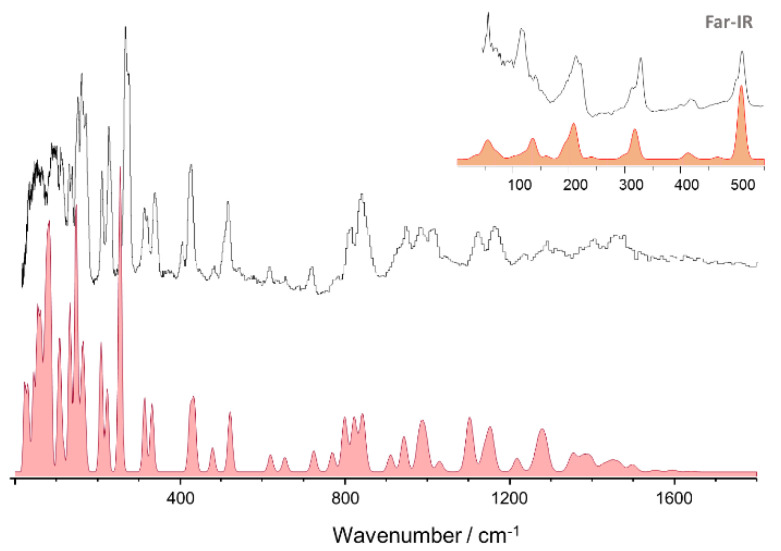
The INS spectra of 4EtOB up to 1800 cm^−1^: experimental (**top**) and simulated from periodic calculations (**bottom**, CASTEP). The theoretical spectra were calculated at the Γ-point and averaged by the dispersion throughout the Brillouin zone (BZ). The full INS spectra, up to 4000 cm^−1^, and the comparison between periodic and discrete calculations at the same level are shown in Figures S1 and S2, Supplementary Material, respectively. The inset presents the Far-IR spectra in the 25–550 cm^−1^ range: experimental (**top**) and simulated from periodic calculations (**bottom**, CASTEP).

It should be stressed that these are not experimental crystal structures; therefore, their validity must be evaluated through the spectroscopic data available, namely, INS and Far-IR spectra. Figure 5 compares the experimental INS spectrum with the one generated from the “fixed cell” crystal structure and the inset depicts the same comparison for the infrared spectra, in the Far-IR region. The “fixed cell” spectrum is presented, since the agreement between the experimental and the predicted spectra for the “relaxed cell” was found to be slightly worse in the low wavenumber region (See Figures S3 and S4, Supplementary Material). For instance, the two bands observed in the INS spectrum at ca. 211 cm^−1^ and 227 cm^−1^ are correctly predicted from the “fixed cell” model, while only one band is predicted from the “relaxed cell” model.

Nevertheless, for the modes above ca. 250 cm^−1^, both models yield identical spectra, and the good correspondence between experimental and calculated spectra in Figure 5 is the basis for the assignments in Table 6.

A tentative assignment of the infrared and Raman spectra of 4-ethoxybenzaldehyde in the liquid phase has been reported by Venkoji et al. [32]. Comparing the reported and present assignments, there is a reasonable qualitative agreement for most of the modes, but with several conflicts in the mid and low frequency regions. For instance, the bands at 1470 cm^−1^ and 1401 cm^−1^ are ascribed to the same modes but in reverse order; the band at 840 cm^−1^, clearly identified as out-of-plane bending of CH in CASTEP calculations (Table 5), is assigned to symmetrical ring CC stretching mode [32]; and the in-plane bending mode of the -CHO group, found at ca. 165 cm^−1^ (Table 5) is assigned to a band at 425 cm^−1^ [32].

**Figure 6:**
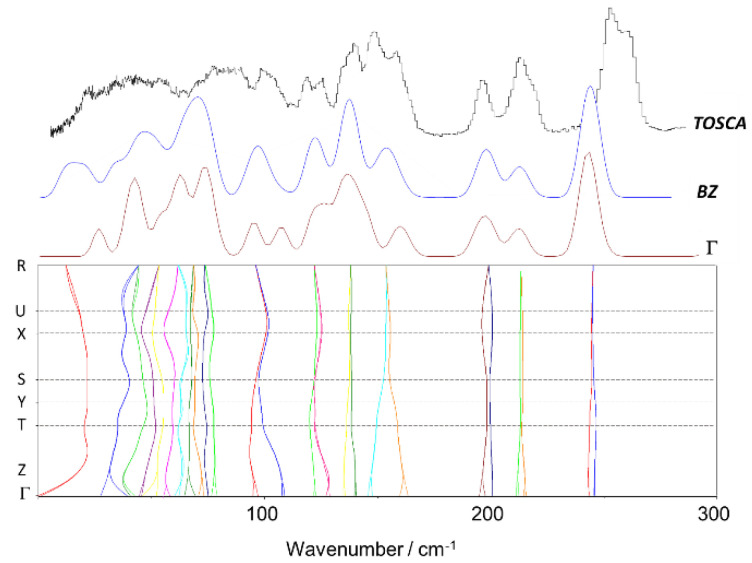
Experimental and calculated (“fixed cell”) INS spectra of 4EtOB in the range below 300 cm^−1^. Bottom panel: calculated phonon dispersion relations along the high symmetry points; top lines: comparison between the INS spectra calculated at the Γ-point (Γ), averaged by the dispersion throughout the Brillouin zone (BZ), and experimental INS spectrum (Tosca).

**Figure 7:**
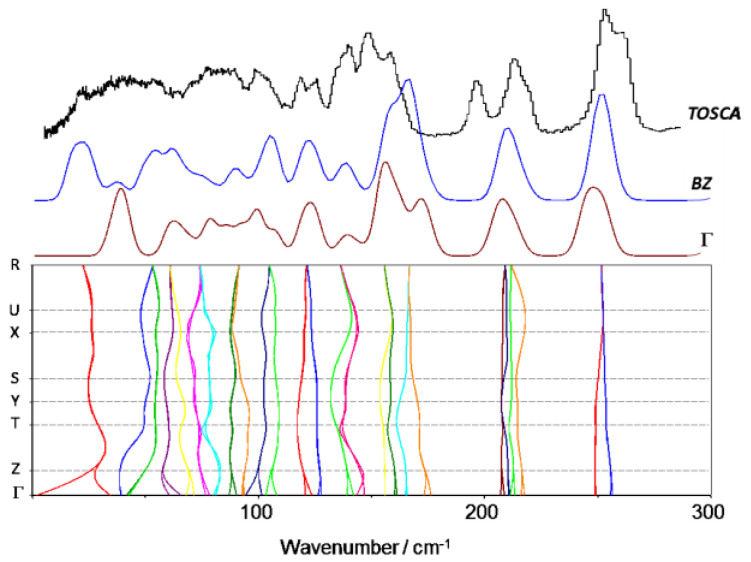
Experimental and calculated (“relaxed cell”) INS spectra of 4EtOB in the range below 300 cm^−1^. Bottom panel: calculated phonon dispersion relations along the high symmetry points; top lines: comparison between the INS spectra calculated at the Γ-point (Γ), averaged by the dispersion throughout the Brillouin zone (BZ), and experimental INS spectrum (Tosca).

Since the optimized crystal structure of 4-ethoxybenzaldehyde is based on the assumption of similarity with 4-methoxybenzaldehyde, it is important to access the phonon dispersion, as it often contains important information about intermolecular interactions in the lattice structure. Unlike infrared and Raman spectra, which are measured at the Γ-point in the Brillouin zone, i.e., at nearly zero wavevector, INS is sensitive to all wavevectors and the vibrational dispersion is directly reflected in the spectrum. Figure 6 and Figure 7 show the phonon dispersion plot for the modes below 300 cm^−1^ of 4-ethoxybenzaldehyde, the calculated INS spectrum at the Γ-point and the calculated INS spectrum averaged by the dispersion across the entire Brillouin zone (BZ), for both calculated crystal structures, “fixed” and “relaxed”, respectively. The dispersion curves for the modes above 300 cm^−1^ are essentially flat, i.e., no measurable dispersion effects are observed above this wavenumber.

Figure 6 and Figure 7 illustrate the dependence of low vibrational modes on phonon dispersion. Modes involving the torsional motions are among the most sensitive modes, as it can be observed from the corresponding dispersion curves. The INS spectra calculated at the Γ-point (Γ) and averaged by the dispersion throughout the Brillouin zone (BZ) for both forms are significantly different in some regions, but none of them provide a clear match with the experimental spectrum. We will turn back to this point in the Discussion section.

### 3.4. Dynamics of Internal Rotors

The potential energy function for hindered internal rotations is generally described by the sum of cosine terms,

V(*θ*) = 1/2 ∑ V*_n_* (1 − cos *n*θ) (1)

where *θ* is the torsional angle, *n* is the periodicity of the rotation, and V*_n_* are the terms contributing to the barrier height. The energy levels for torsional motion can be derived by solving the Hamiltonian for the internal rotor with known barrier, or the barrier height can be estimated by fitting the energy level differences to the observed torsional transitions [26].

Among the different dynamical processes that take place in the herein presented crystals, methyl group rotation deserves special attention. Methyl torsion can be addressed as a nearly pure normal mode and all relevant interactions on the methyl group can be condensed in an effective one-dimensional potential.

**Figure 8:**
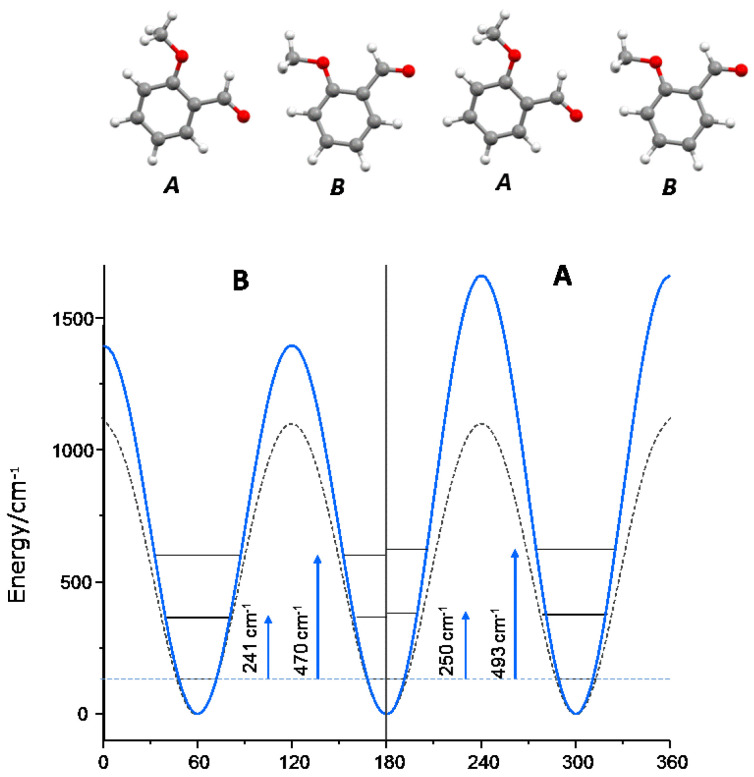
Top: sequence of A–B–A–B type molecules in the crystal structure of 2MeOB, evidencing the different methyl group neighboring. Bottom: potential energy function for (**B**) a (**A**) molecules derived from observed torsional frequencies (solid lines) and calculated for the isolated molecule (dashed line).

The 2MeOB crystal presents sixteen molecules in the unit cell, and therefore there are sixteen torsional transition energies for methyl torsion. These transitions pack together in two strong INS bands, as observed experimentally and well predicted from calculations (see Table 2). From the visualization of atomic displacements for each torsional mode, the two bands can be assigned to specific molecules in the crystal: the lower wavenumber band is related with the methyl groups of the B/D-type molecules, while the higher wavenumber component arises from the methyl groups of A/C-type molecules (Figure 8, top panel). Figure 8, bottom plot, shows the two potential energy functions derived from the experimental values (solid lines), compared with the calculated potential energy function for the isolated molecule, obtained from a relaxed geometry scan (dashed line). The calculated V_3_ potential for the isolated molecule (1107 cm^−1^) compares with the calculated values for anisole derivatives at the MP2/6–311++G(d,p) level (e.g., 1118 cm^−1^ [33] and 1035 cm^−1^ [34]).

4MeOB has a single torsional band resulting from four identical methyl groups in the unit cell. The methyl group dynamics in 4MeOB is not quite different from the one observed for the B-type molecules in 2MeOB, with a V_3_ potential of ca. 1400 cm^−1^. The calculated V_3_ methyl rotation potential for the isolated molecule (1055 cm^−1^) is somewhat higher than the experimental value reported from the gas phase millimeter-wave absorption spectrum (804 cm^−1^, [35]).

A different situation arises for the methyl group in 4EtOB, for which the internal rotation has a large V_6_ contribution, which must stem from relevant intermolecular interactions in the crystal. The calculated rotational potential energy for the isolated molecules is a pure V_3_ = 1079 cm^−1^.

These results are summarized in Table 7.

It should be mentioned that the potential energy terms for the formyl (aldehyde) and methoxy/ethoxy groups can also be obtained from the torsional frequencies assigned in Table 2, Table 4 and Table 6. For the -CHO torsion, pure V_2_ terms of ca. 3300 cm^−1^, 2800 cm^−1^, and 2300 cm^−1^ are obtained for 2MeO, 4MeOB, and 4EtOB, respectively. For the alkoxy group rotation, corresponding V_2_ values are 4600 cm^−1^, 5400 cm^−1^, and 5300 cm^−1^, in the same order. For 4MeOB, both values are well above those reported for the gas phase [35,36], suggesting that crystal packing plays a significant role in the torsional barrier. However, these barrier values are only indicative, as calculations assume pure torsional modes, which is not the case for these rotors: analyses of the calculated normal modes reveal a significant mixing between the torsions of methoxy and CHO groups, as discussed below.

## 4. Discussion

The vibrational assignments comprised in Table 2, Table 4, and Table 6 strongly support the reliability of CASTEP calculations [1,7,9,22] for these type of systems, as well as the tight correlation between the intensity of each INS band and the atomic displacements of the vibrational mode.

Periodic calculations are found to be of utmost importance in the description of the vibrational spectra of these systems, which are mostly van der Waals crystals with weak C–H...O contacts. The single molecule approach leads to the defective description of several low wavenumber vibrational modes, as mentioned in previous sections. This is particularly evident in the direct comparison between discrete and periodic predicted INS spectra in the wavenumber region below ca. 300 cm^−1^ (Figure S2). Apart from the obvious missing of the collective modes, single molecule spectra fail to properly describe large amplitude vibrational modes, such as C–OCH_3_ torsion and C–O–C bending modes, among other.

One point deserving attention is the presence of two bands associated with the same vibrational mode, identified in Table 2 (2MeOB) and 6 (4EtOB). These band splittings are mainly observed in the large amplitude/low wavenumber modes, an observation that relates them with intermolecular interactions in the crystal.

In the case of 2MeOB, the pairs of bands are easily assigned to the independent molecules in the asymmetric unit. The band splittings are correctly predicted by CASTEP calculations, and visualization of atomic displacements clearly identifies the bands belonging to A/C- or B/D-type molecules in the crystal (Table 2).

In what concerns 4EtOB, the observed band splitting cases are not predicted from CASTEP calculations and must stem from discrepancies between the experimental sample and its CASTEP model. This may arise from (i) the incompleteness of the model, with a single independent molecule in the asymmetric unit (as derived from 4MeOB X-ray structure), or (ii) the conformational disorder of the ethoxy-group/aldehyde group in the crystal. Although none of these can be easily ruled out from the data available, there are some hints towards the incompleteness of the model. Namely, the similarity between the observed shifts in 4EtOB and 2MeOB and the absence of band splitting in higher wavenumber modes, which are to be expected in the case of conformational disorder of the ethoxy group.

It should be mentioned that for 4MeOB the presence of pairs of bands in the infrared and Raman spectra has been reported for the liquid phase. These pairs were undoubtedly related with the anti-syn conformational equilibrium in the liquid phase [29] and are absent from the spectra of the solid phase. None of the bands belonging to the less stable conformer (syn form) is observed in the INS spectrum of 4MeOB. The small “non-fundamental” bands observed along the spectra (e.g., 311, 368, 444 and 540 cm^−1^, see Figure 3) are easily identified as overtones and combination bands from AbINS simulation.

Another question is related with the torsional motions of substituent groups, such as methyl, aldehydic, and alkoxy groups. The option for a simplified approximate description of the normal modes in Table 2, Table 4, and Table 6—less rigorous but more readable than the often cumbersome P.E.D. description, as already discussed elsewhere [1]—is not intended to hide the complex nature of some vibrational modes. Besides the modes belonging to the aromatic ring, whose conventional description is well-known, for most of the low-wavenumber vibrations involving the substituent groups, the single oscillator mode is more an exception than a rule. For instance, strong coupling between the torsional modes of aldehyde and alkoxy groups is observed. Their labeling on Table 2, Table 4, and Table 6 identifies the most relevant contribution, but none of these modes can be described as a pure torsion. A different situation arises for the torsion of the methyl groups, which was found to be highly localized and can be addressed as a nearly pure mode. This is relevant because INS spectroscopy is the only technique that allows observing the torsional transitions of methyl groups, as it overcomes the limitations of optical spectroscopy: INS provides ready access to the low wavenumber region and is not hampered by selection rules and low intensity of the torsional modes. In this way, it is possible to describe the potential energy function for the internal rotation of methyl groups in terms of its V_3_ and V_6_ components (Table 7) from INS spectra.

It has been shown that the torsional modes of methyl groups bonded to sp^2^ carbon atoms occur at ≤200 cm^−1^ while those bonded to sp^3^ carbon atoms appear at ~250 cm^−1^ [37]. The present results for 2MeOB and 4MeOB show that methyl groups bonded to (sp^3^) oxygen atoms also occur at ~250 cm^−1^, thus pointing to a similar nature of the potential energy barrier for the rotation along the saturated C–CH3 and O–CH3 bonds.

The importance of intermolecular interactions in the torsional potential can also be drawn from present results. As mentioned above, the large V_6_ component for methyl rotation of 4EtOB is not present in the isolated molecule and must stem from intermolecular interactions. In the case of 2MeOB, the A/C-type methyl groups, with shorter intermolecular CH_3_···O=C contacts, display a V_3_ torsional barrier ca. 270 cm^−1^ higher than the B/D-type methyl groups. This means that the CH···O intermolecular contacts represent at least 17% of the barrier height for methyl torsion in A/C-type molecules. However, taking the reference of the calculated value for the isolated molecule, the barrier increase due to intermolecular contacts for these molecules (ca. 550 cm^−1^) is 1/3 of the final barrier height.

The comparisons in Figure 5 and Figure 6 suggest that the optimized periodic structure of 4EtOB is a good approach to the crystal structure. But can it be assumed as the real structure, with a good level of confidence? As noted elsewhere [4,10], a good match between experimental and calculated spectra INS provides straightforward validation of the model. This is particularly important for the low wavenumber region—where the external modes dominate and, thus, the sensibility to crystal packing is higher. However, this region is the poorly described region in CASTEP calculations. Important discrepancies between calculated and experimental spectra in the region of the intermolecular modes are reported for several systems, even when the actual crystal structure is well known [7,37,38]. These discrepancies are a natural result from limitations of the periodic calculations (e.g., harmonic oscillator approximation, incomplete description of dispersion interactions, energy cut-offs, and the sum of numerical errors, which accumulate in the low wavenumber modes). As stated elsewhere [38]: “DFT still has some way to go before it can predict intermolecular modes with the same reliability that it does for intramolecular modes”.

Careful inspection of the low wavenumber region in Figure 6 and Figure 7 reveals a very reasonable but not excellent agreement between calculated and experimental spectra. Significantly, the fully optimized (“relaxed cell”) model does not provide a clearly better description of this critical region, when compared with the “fixed cell” model. For the vibrational modes that can be described as purely intermolecular, e.g., below 100 cm^−1^, none of the calculated spectra is fully satisfactory.

## 5. Conclusions

The excellent agreement between calculated and experimental INS spectra provides a good basis for interpreting the vibrational spectra and assessing the structure and dynamics of the title compounds in the crystal form. The molecular (internal) modes are better described than the collective (external) modes, a well-known drawback of periodic calculations. Nevertheless, several spectral features are unambiguously assigned and torsional potential barriers for the methyl groups are obtained. The intramolecular nature of the potential energy barrier about O–CH_3_ bonds compares with the one reported for torsion about saturated C–CH_3_ bonds, while the intermolecular contribution to the potential energy barrier may represent 1/3 of the barrier height.

On the whole, the model crystal structure proposed for 4EtOB fails for important reasons (such as the splitting of the methyl group torsional mode) and it is not unambiguous concerning the description of the collective modes. However, it provides a good description of the vibrational spectra for a large number of spectral features—including the Far-IR region—allowing a reliable assignment of the INS spectrum.

## Figures and Tables

**Table 1:** Selected geometrical parameters for 2MeOB.

Parameter	X-ray [13]	CASTEP
Bond length/pm		
C7=O1	121.1	123.2
C2–O2	135.8	135.8
O2–C8	142.8	143.5
Bond angle/°		
C1–C7=O1	124.1	124.2
C2–O2–C8	118.6	117.8
Dihedral angle/°		
C6C1–C7O1	−5.4	−3.3
C1C2–O2C8	−178.7	−177.8
C-H…O distance/pm		
C3(A)…O1(B)	347.4	336.3

**Table 2:** Experimental and calculated INS maxima for 2MeOB with mode assignment.

Calculated/cm^–1^ (CASTEP) ^1^	Experimental/cm^–1^ (TOSCA)	Approximate Description
3105	3070	νCH
1440	1435	βCH_3_
1382	1384	βCH(=O)
1277	1279	βCH
1235	1232	βCH
1184	1176	βCH
1135–1145	1145	βCH+rock CH_3_
1091	1088	βCH
1031	1031	βCH
1015	1002	νO–CH_3_
991	983	γCH(=O)
956	951	γCH
867	865	γCH
828	830	αα ring
770	766	γCH
724	718	δ ring
638	640	α ring
575	575	α ring
526	527	δ ring
472	475	α ring
445	438	δ ring
396	399	β ring–OCH_3_
285	290	γ ring–OCH_3_
270	280	β C–O–C (A)
263	267	β C–O–C (B)
238	250	τ O–CH_3_ (A)
228	241	τ O–CH_3_ (B)
200	208	β ring–CHO
168	178	γ ring–CHO (A)
162	172	γ ring–CHO (B)
156	160	τ C–OCH_3_ (A)
146	148	τ C–OCH_3_ (B)
140	139	τ C–CHO (B)
132	126	τ C–CHO (A)
91	98	Libration
67	81	Libration
50	70	Translation
40	62	Translation

^1^ Maxima in the INS simulated spectrum. ν, α, β, γ, and τ stand for stretching, in-plane deformation, out-of-plane deformation and torsion modes, respectively.

**Table 3:** Selected geometrical parameters for 4MeOB.

Parameter	X-ray [14]	CASTEP
Bond length/pm		
C7=O1	121.4	123.0
C2–O2	135.7	135.3
O2–C8	142.5	144.0
Bond angle/°		
C1–C7=O1	126.5	126.2
C4–O2-C8	118.0	117.6
Dihedral angle/°		
C6C1–C7O1	4.3	3.1
C3C4–O2C8	−2.9	−2.8
C-H…O distance/pm		
C3(H)…O2	360.3	336.2
C5(H)…O1	344.7	335.7
C8(HA)…O1	358.2	350.9
C8(HB)…O1	343.4	337.0

**Table 4:** Experimental and calculated INS maxima for 4MeOB with mode assignment.

Calculated/cm^–1^ (CASTEP) ^1^	Experimental/cm^–1^ (TOSCA)	Approximate Description
3110	3096	νCH
1496	1508	βCH
1446	1451	βCH_3_
1387	1396	βCH(=O)
1287	1304	βCH
1156	1170	βCH
1097	1110	βCH
986	1004	γCH(=O)
970	977	γCH
941	948	γCH
837	840	γCH
806	827	γCH
753	760	νCC
719	717	δ ring
636	644	α ring
600	605	α ring
513	518	δ ring
481	479	β ring–OCH_3_
425	426	δ ring
392	395	α ring
329	335	γ ring–OCH_3_
269	273	β C–O–C
245	247	τ O–CH_3_
200	205	γ ring–CHO
183	190	β ring–CHO
165	160	τ C–OCH_3_
121	128	τ C–CHO
100	105	Libration
88	90	Libration
80	75	Libration
69	67	Libration
54	51	Translation
49	39	Translation

^1^ Maxima in the INS simulated spectrum. ν, α, β, γ, and τ stand for stretching, in-plane deformation, out-of-plane deformation and torsion modes, respectively.

**Table 5:** Selected geometrical parameters for 4EtOB.

Parameter	Fixed Cell	Relaxed Cell
Cell dimensions/pm		
a	560	616.2
b	903.4	892.6
c	1750	1442.6
Bond length/pm		
C7=O1	122.8	122.8
C4–O2	135.4	135.2
O2–C8	145.1	145.2
Bond angle/°		
C1–C7=O1	126.3	126.2
C4–O2–C8	118.1	117.8
Dihedral angle/°		
C6C1–C7O1	1.8	4.5
C3C4–O2C8	−2.3	−4.1
C-H…O distance/pm		
C5(H)…O1	341.3	339.3
C9(H)…O1	342.8	352.1
C8(HB)…O2	400.0	392.0

**Table 6:** Experimental and calculated INS maxima for 4EtOB with mode assignment.

Calculated/cm^–1^ (CASTEP) ^1^	Experimental/cm^–1^ (TOSCA)	Approximate Description
3141	3124	νCH
1440	1470	βCH_3_
1381	1401	βCH(=O)
1346	1375	δCH_2_ wag
1266	1293	δCH_2_ twist
1208	1236	νC–CHO
1140	1165	βCH
1094	1123	βCH
1021	1044	νO–CH_2_CH_3_
979	1016	γCH(=O)
933	985	γ CH
900	950	γ CH
832	840	γ CH
792	812	γ CH
760	780	νCC
714	720	δ ring
647	655	α ring
611	618	α ring
511	519	δ ring
471	482	β ring–OCH_2_CH_3_
421	426	δ ring
322	339	γ ring–OCH_2_CH_3_
304	315	β O–C–C
245	268/274	τ O–CH3
214	227/233	β C–O–C
199	211	γ ring–CHO
155	161/170	β ring–CHO
138	152	τ C–OCH_2_CH_3_
123	131/137	τ O–CH_2_CH_3_
98	115	τ C–CHO
71	96	Libration
49	61	Libration
35	46	Translation
17	34	Translation

^1^ Maxima in the INS simulated spectrum. ν, α, β, γ, and τ stand for stretching, in-plane deformation, out-of-plane deformation and torsion modes, respectively.

**Table 7:** Components of the potential energy function for methyl rotation, derived from torsional frequencies. All values are in cm^−1^.

	2MeOB (B/D)	(A/C)	4MeOB	4EtOB
0–1	241	250	247	267
0–2	470	493	480	508
V_3_	470	493	480	508
V_6_	−16	−73	3	147
V_3_ isolated (G09)	1107		1055	1079
V_3_ gas phase			804 [35]	

## Data Availability

Not applicable.

## Supplementary Materials

The following are available online at https://www.mdpi.com/article/10.3390/ma14164561/s1, Figure S1: INS spectra of title compounds up to 4000 cm^−1^. Figure S2: Calculated INS spectra of title compounds from discrete (G09) and periodic (CASTEP) calculations; Figure S3: Experimental and calculated INS spectra for 4-ethoxybenzaldehyde. Figure S4: Experimental and calculated Far-IR spectra for 4-ethoxybenzaldehyde. CIF files for the optimized CASTEP structures of 4-ethoxybenzaldehyde.
