# Supplementary material for: New Insights on the Vibrational Dynamics of 2-Methoxy-, 4-Methoxy- and 4-Ethoxy-Benzaldehyde from INS Spectra and Periodic DFT Calculations
Source: Materials (Basel). 2021 Aug 13;14(16):4561. doi: 10.3390/ma14164561 (PMC8399430; doi:10.3390/ma14164561)

New insights on the vibrational dynamics of 2-methoxy-, 4-methoxy- and 4-ethoxy-benzaldehyde from INS spectra and periodic DFT calculations

Paulo J.A. Ribeiro-Claro [1], Pedro D. Vaz [2], Mariela Nolasco [1,*], Francisco P.S.C. Gil [3], Luís A.E. Batista de Carvalho [4], M. Paula M. Marques [4,5] and Ana M. Amado [4]

Supplementary Material

Figure S1. *INS spectra of 2-methoxybenzaldehyde (a), 4-methoxybenzaldehyde (b) and 4-ethoxybenzaldehyde (c) up to 4000 cm*$^{-1}$.

Figure S2. *Discrete (G09) and periodic (CASTEP) INS spectra of 2-methoxybenzaldehyde (a), 4-methoxybenzaldehyde (b) and 4-ethoxybenzaldehyde (c) up to 900 cm*$^{-1}$*, compared with the experimental spectrum.*

Figure S3. *Experimental and calculated INS spectra for 4-ethoxybenzaldehyde*

Figure S4. *Experimental and calculated Far-IR spectra for 4-ethoxybenzaldehyde*

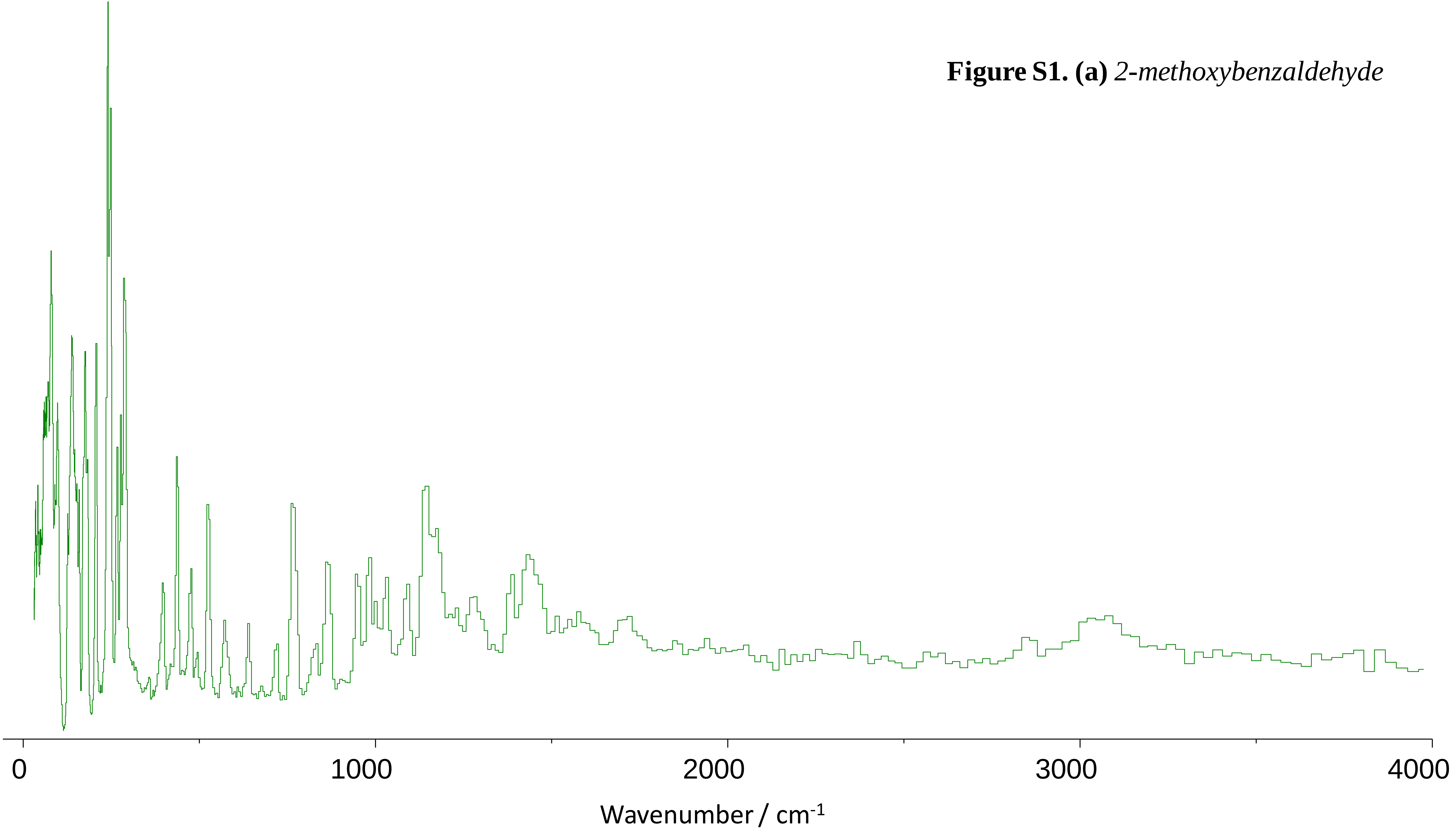

Figure S1. (a) *2-methoxybenzaldehyde*

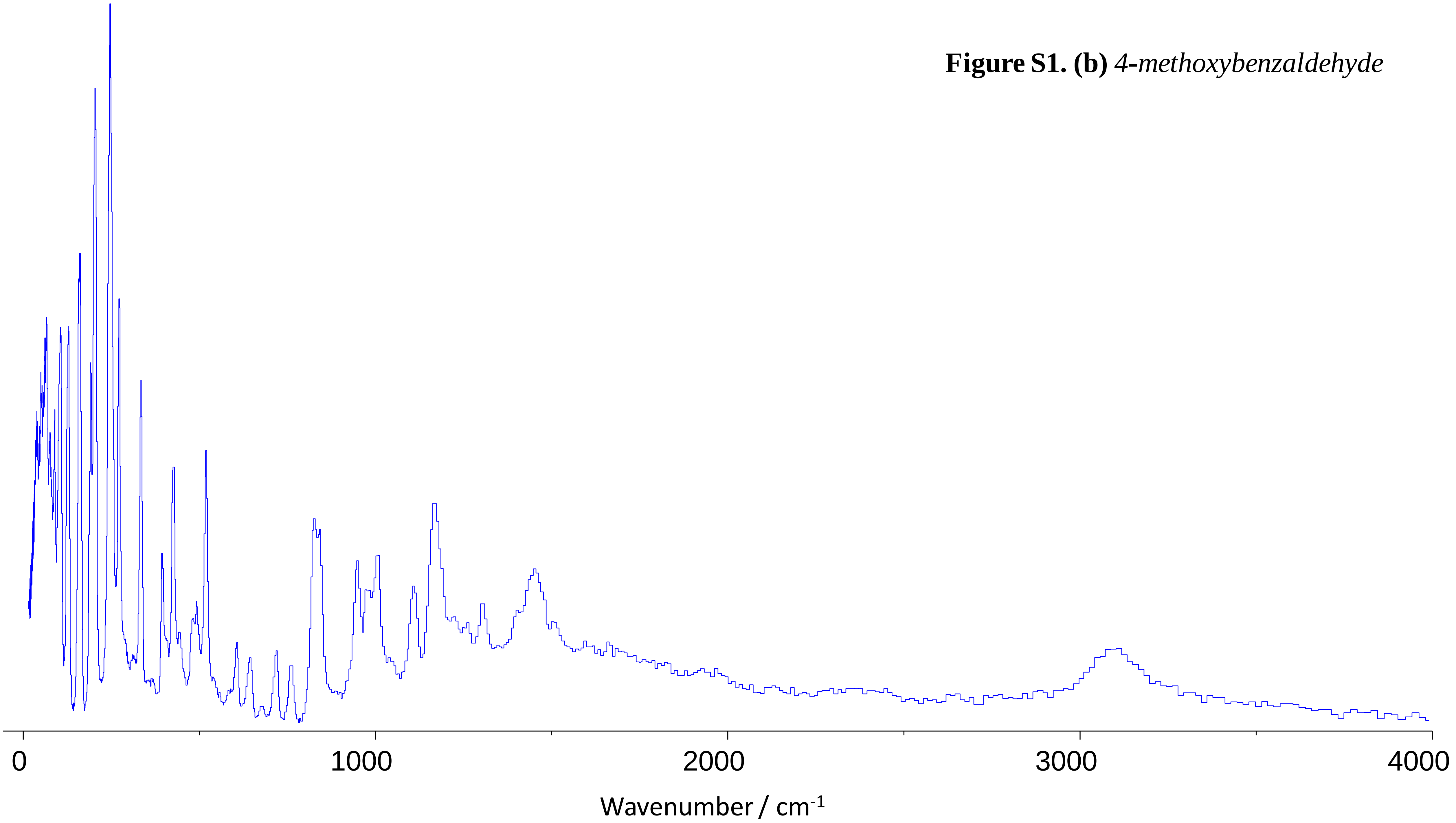

Figure S1. (b) *4-methoxybenzaldehyde*

Figure S1. (c) *4-ethoxybenzaldehyde*

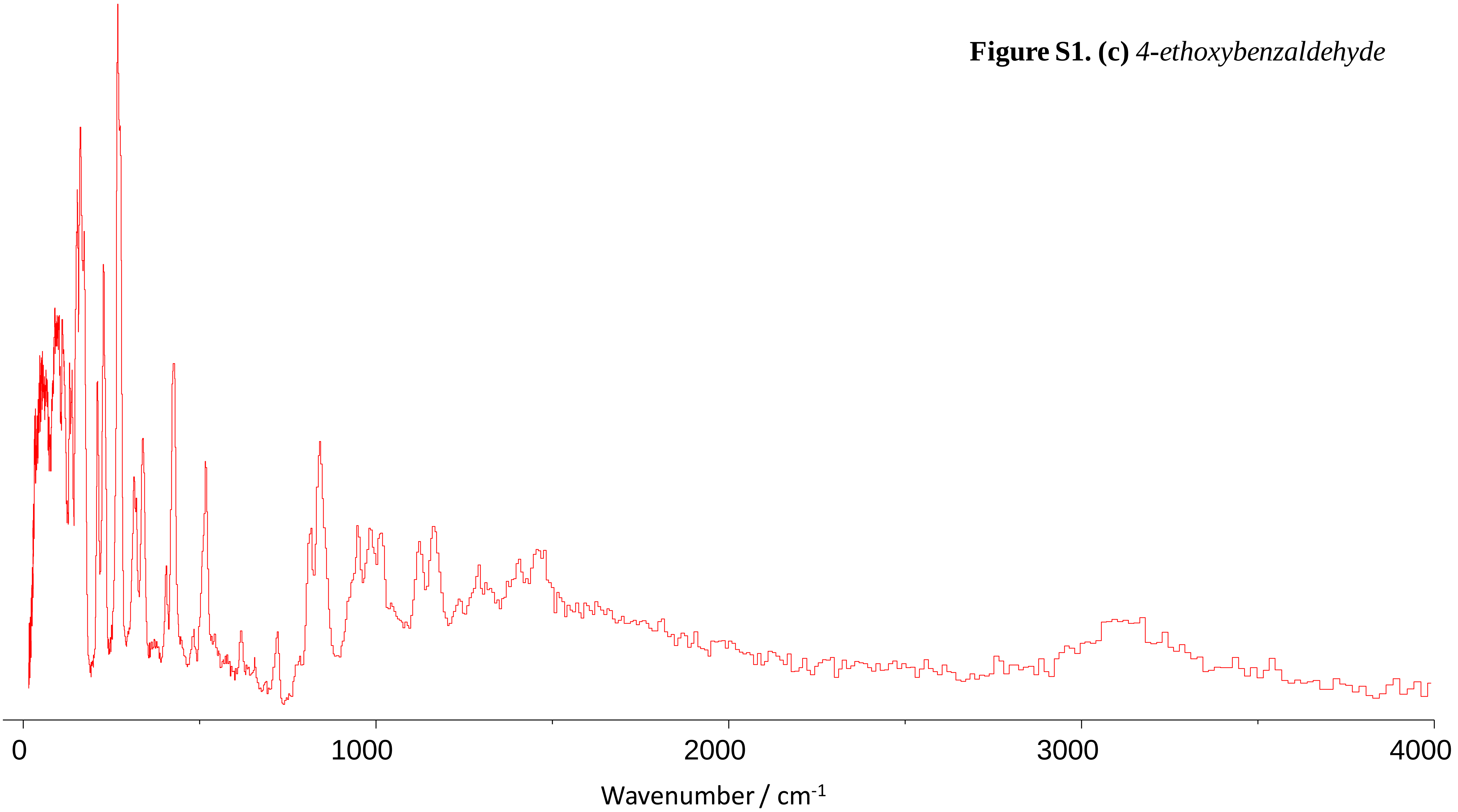

Figure S2. (a) *2-methoxybenzaldehyde*

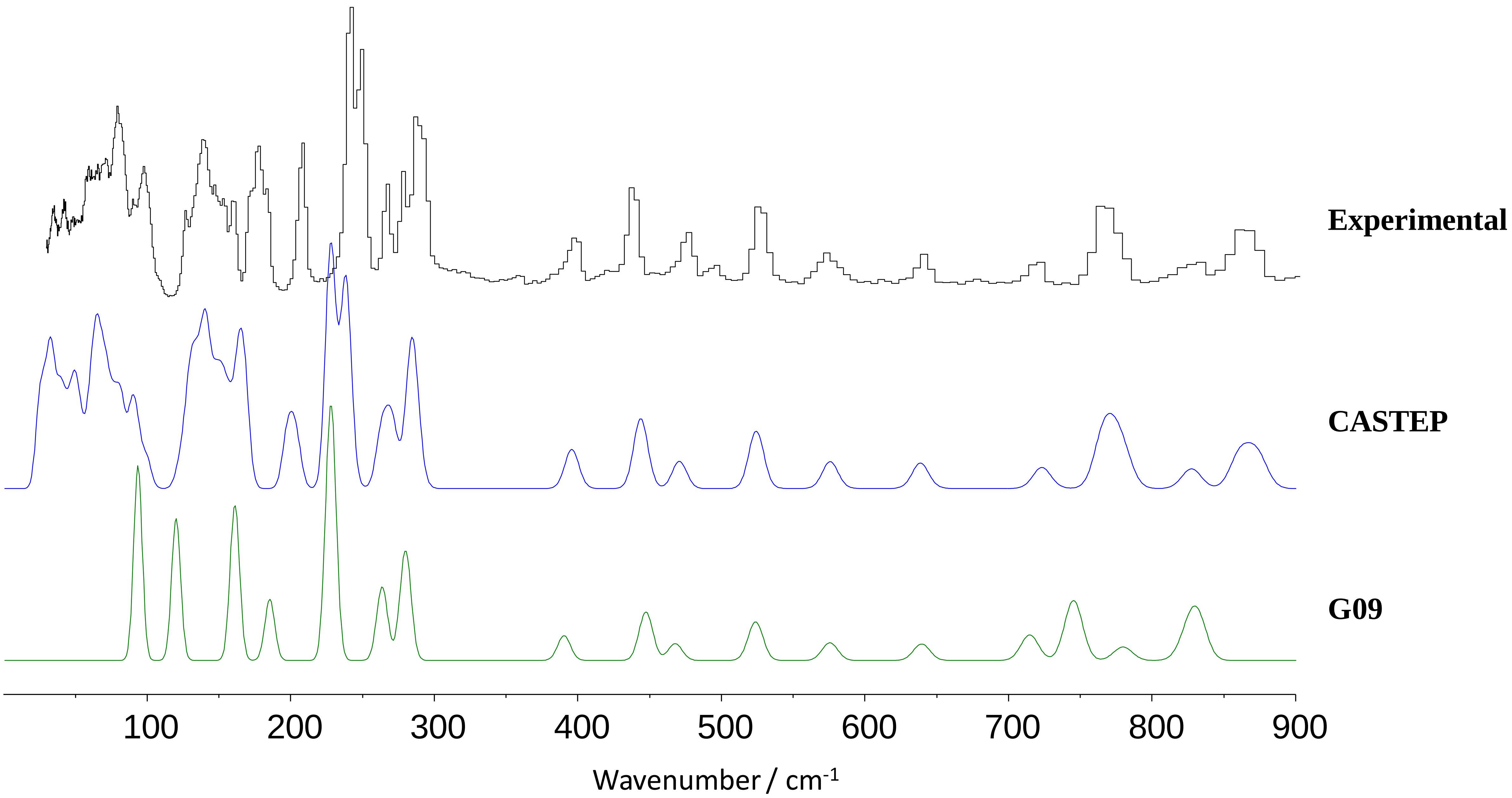

Figure S2. (b) *4-methoxybenzaldehyde*

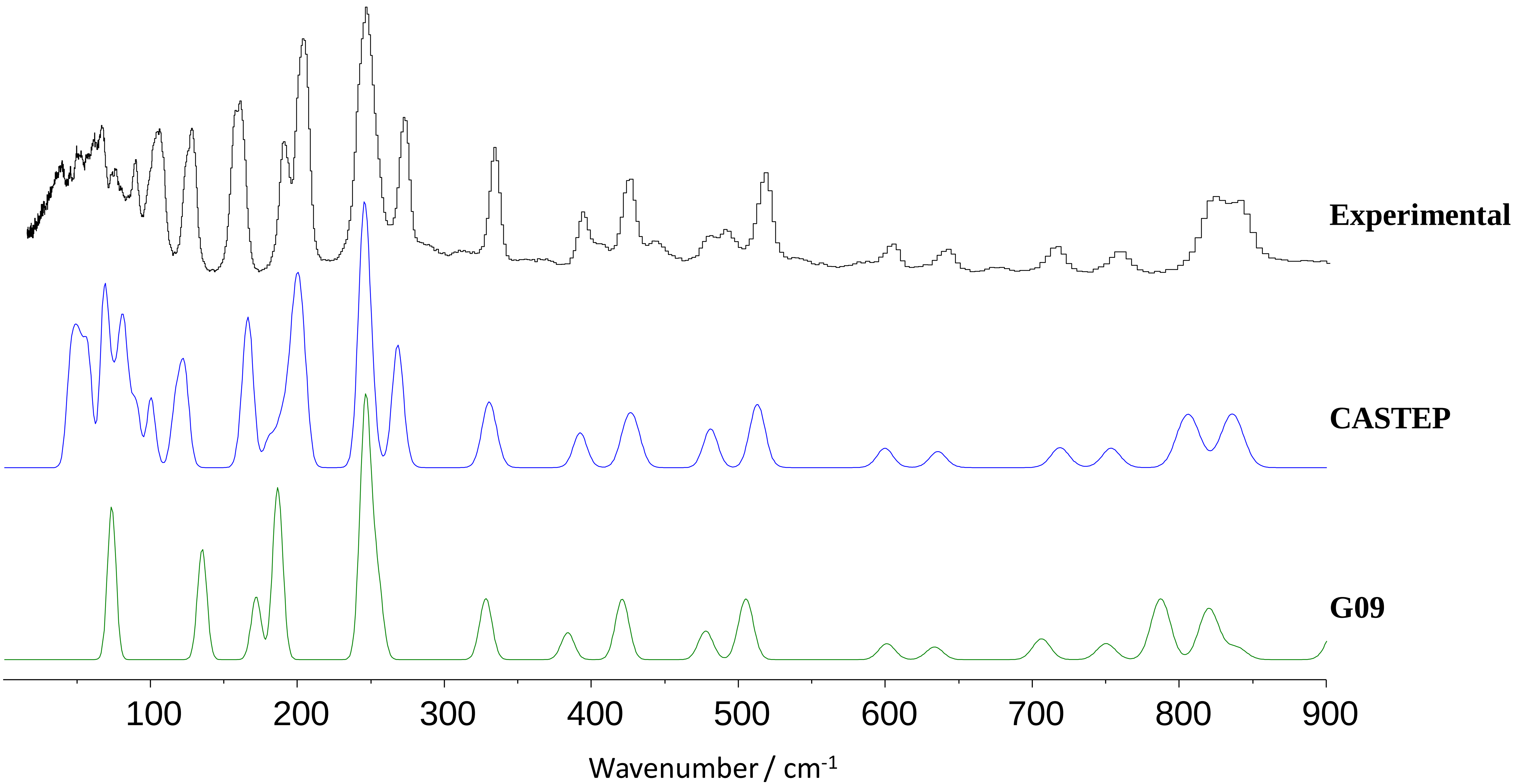

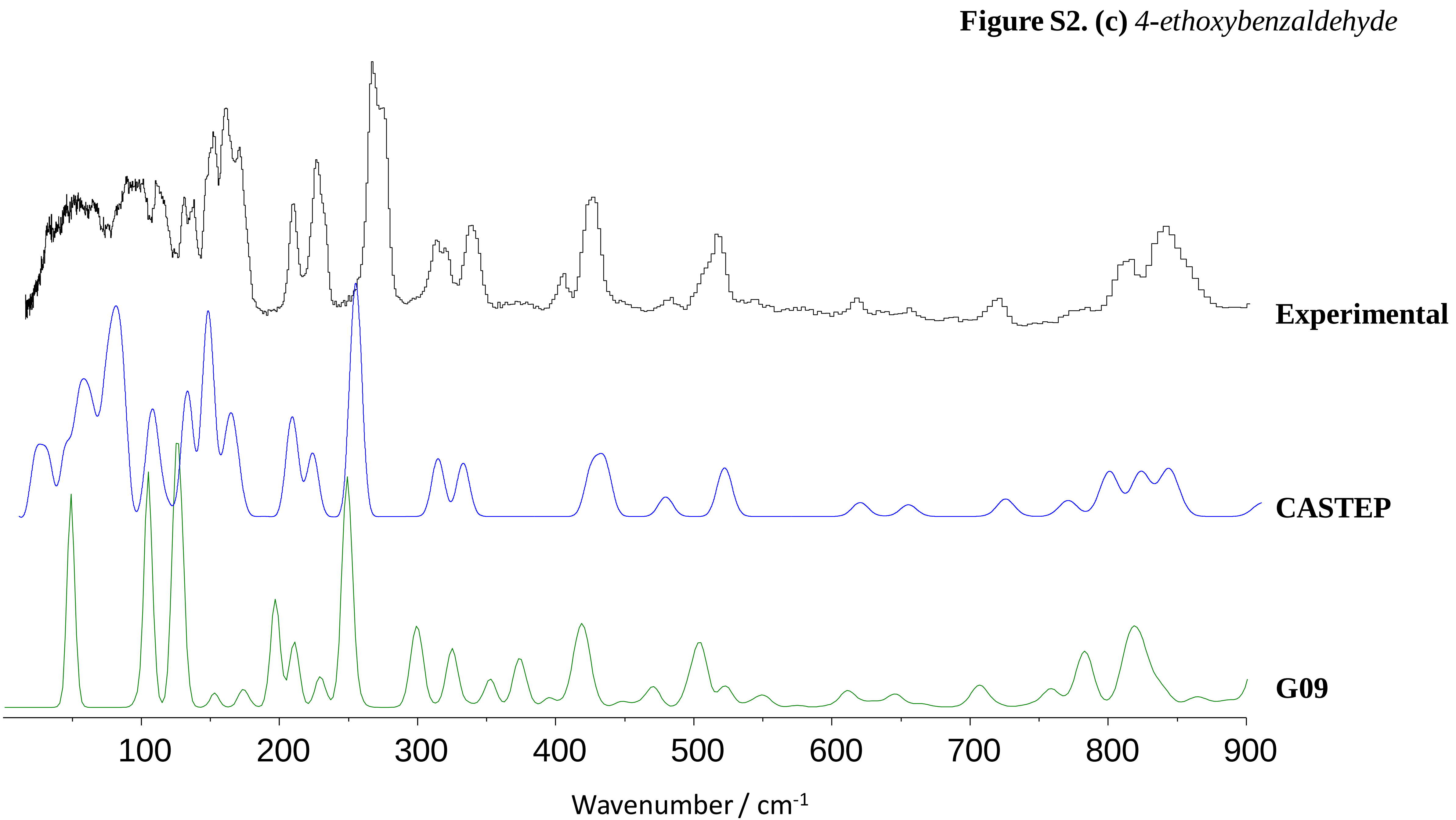
Figure S2. (c) 4-ethoxybenzaldehyde
Experimental
CASTEP
G09
100
200
300
400
500
600
700
800
900
Wavenumber / cm-1

Figure S3. *Experimental and calculated INS spectra for 4-ethoxybenzaldehyde*

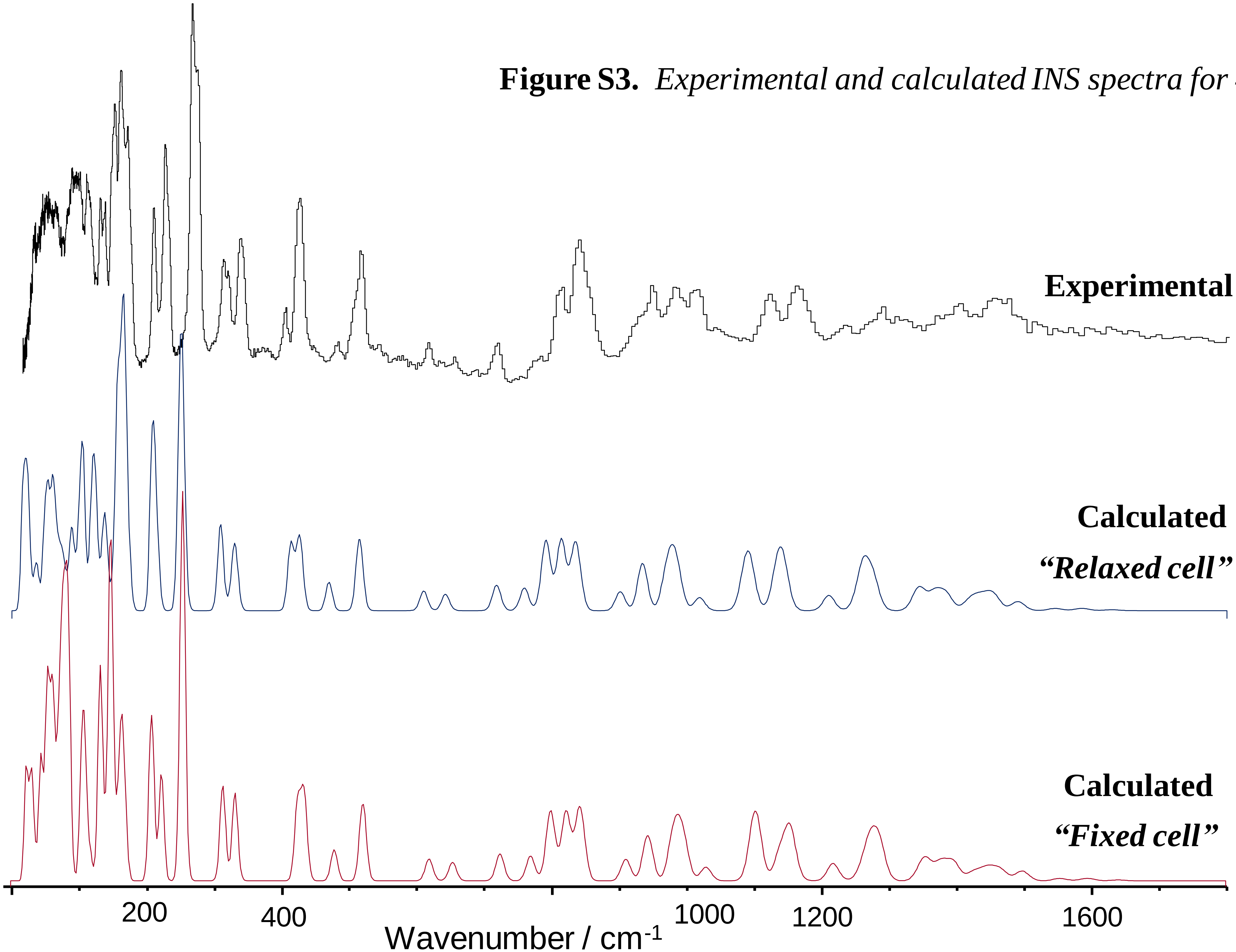

Figure S4. *Experimental and calculated Far-IR spectra for 4-ethoxybenzaldehyde*

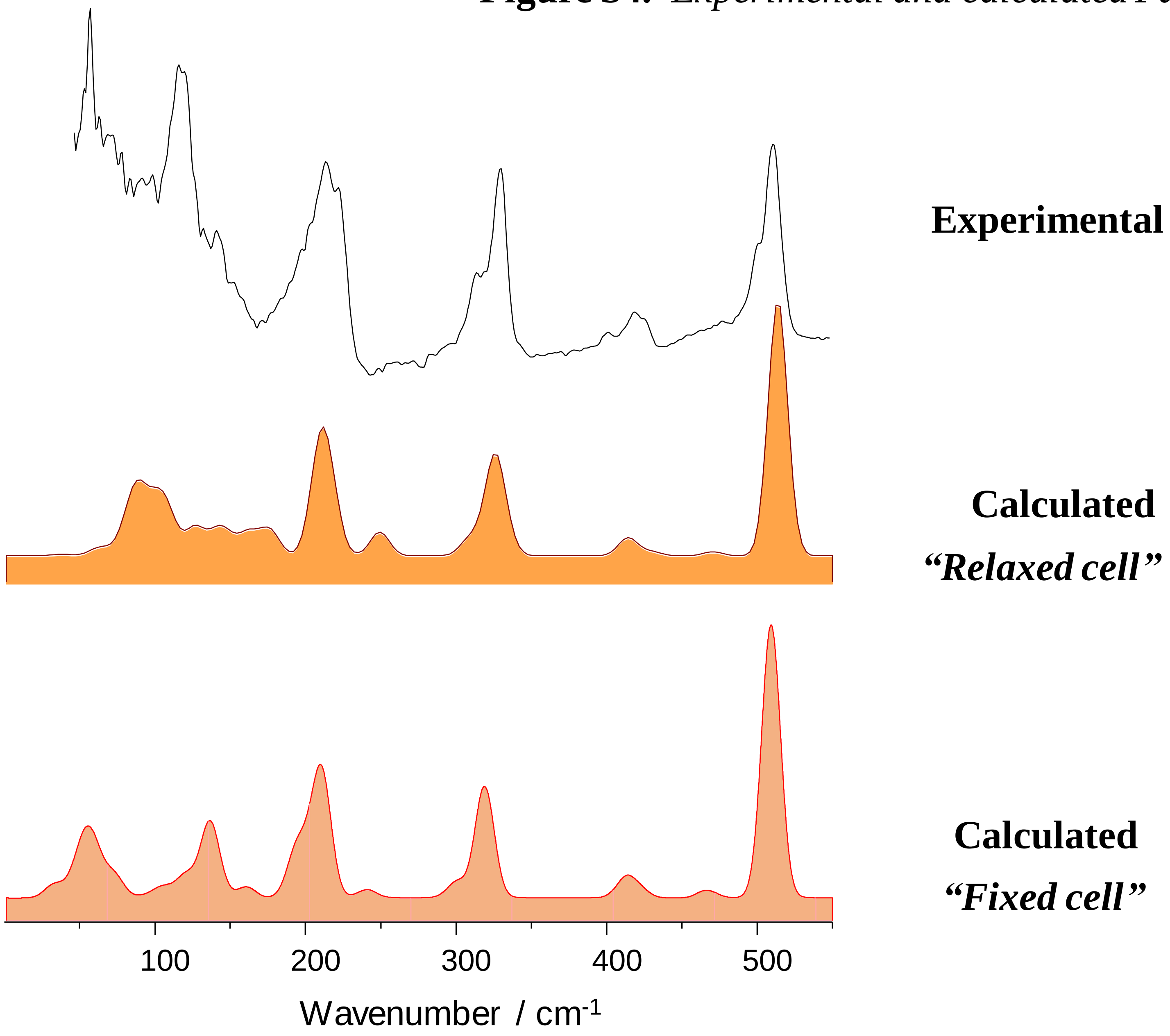

Supplement: Supplementary file 1 [file materials-14-04561-s001.zip › ESIRev2.pdf]
